# Mitigating Resistance to Malaria Treatments in Sub-Saharan Africa Requires More than New Drugs

**DOI:** 10.4269/ajtmh.24-0781

**Published:** 2025-02-25

**Authors:** Lawrence M. Barat

**Affiliations:** Independent Consultant, Provincetown, Massachusetts

## Abstract

In September 2024, the US President’s Malaria Initiative; the Global Fund for AIDS, Tuberculosis, and Malaria; the Gates Foundation; and Unitaid called for malaria partners to increase the availability and lower the cost of alternative artemisinin-based combination therapies (ACTs) for countries with growing evidence of resistance to artemisinin and current ACT partner drugs, particularly in sub-Saharan Africa. Although these global leaders should be applauded for raising this challenge to the highest levels, they missed the opportunity to highlight a major driver of resistance to malaria treatments: the limited access to high-quality health services for malaria. Progress has been made in scaling up integrated community case management and clinical and laboratory quality improvement programs, but few malaria-affected countries have achieved national scale. If affected countries and their partners do not want to confront resistance to these newer alternative ACTs in the near future, they must take more decisive action now to expand access and improve the quality of malaria services.

In September 2024, the US President’s Malaria Initiative (PMI); the Gates Foundation; the Global Fund for AIDS, Tuberculosis, and Malaria (Global Fund); and Unitaid issued a “Donor Statement on Urgent Action Required to Address Antimalarial Drug Resistance,”^1^ highlighting a serious issue: the emergence of partial resistance to artemisinins and early evidence of artemisinin-based combination therapy (ACT) partner drug resistance in some countries in sub-Saharan Africa (SSA). In particular, early signs of possible resistance to lumefantrine, a component of the ACT artemether-lumefantrine (AL), are of great concern because AL is the first-line treatment for malaria in the majority of countries in SSA.^1^

The statement focused on the lack of availability and high cost of alternative ACTs, including dihydroartemisinin-piperaquine and pyronaridine-artesunate. These alternative ACTs have the potential to replace current first-line ACTs (AL and artesunate-amodiaquine) in most countries in SSA or could be adopted as second-line treatments. The statement was launched alongside the 79th UN General Assembly, calling on other donors, foundations, and the private sector to collaborate in reducing the cost and expanding the availability of alternative ACTs.^2^

These partners should be commended for bringing attention to the challenges of emerging resistance to malaria treatments in SSA. However, this call to action missed an important opportunity to highlight systemwide challenges that have contributed and will continue to contribute to the spread and intensification of artemisinin and partner drug resistance in SSA. Unlike many preventive strategies, which are primarily delivered through campaigns that often circumvent bottlenecks in national health systems, the diagnosis and treatment of clinical malaria must be provided through these health systems, which, despite significant improvements in recent years, remain limited in scale and quality, and vary in their points of delivery. The WHO 2024 health statistics score SSA’s access to 14 core health services, known as the Universal Health Coverage (UHC) service coverage index, at only 44 out of 100, highlighting that access to essential healthcare services is limited for most of its population.^3^

Efforts to increase access to basic services through the introduction of integrated community case management (iCCM) have had some success in improving access to care in targeted areas. However, a 2021 analysis of Demographic and Health Survey data estimated that although one-quarter of sick children received care from a community health worker (CHW) in areas where they are present, only 2% of sick child care was provided by CHWs. This discrepancy is largely due to the fact that CHW programs have not achieved scale in most countries.^4^

A recent special supplement to this journal highlighted the successes of PMI’s investments in the quality improvement (QI) of clinical and laboratory services for malaria through the implementation of the Outreach Training and Supportive Supervision (OTSS) approach. However, OTSS has only been implemented on a national scale in a handful of countries, most notably Ghana and Zambia.^5^^,^^6^

Despite private sector outlets providing a median of 28% of all malaria diagnosis and treatment services in SSA (exceeding 50% in some countries), little to no investment has been made to improve the quality of malaria services in the private sector beyond a few time-limited pilots.^7^^,^^8^ Not surprisingly, a comprehensive review by Goodman et al.^9^ found no evidence of the large-scale implementation of malaria rapid diagnostic tests or iCCM in the private sector. The diversity of private sector outlets, which range from private hospitals and clinics to pharmacies, chemical sellers, and general stores, creates unique challenges to improving quality. Data from the ACTWatch Lite project in Benin and Cameroon, for example, found that 7% and 46%, respectively, of outlets stocking malaria treatments were general stores, whereas 45% and 31%, respectively, were for-profit health facilities.^10^^,^^11^ Given this diversity, tailored QI approaches and implementation platforms are needed for different private provider cadres.

Compounding the barriers to access and quality clinical services, national supply chains for essential medicines, although improved over the last 20 years, still face challenges in maintaining consistent supplies of malaria diagnostics and treatments at the point of care. Funding for these commodities, although markedly improved through increased country budgets and support from PMI and the Global Fund, still does not fully meet country needs. Additionally, the logistics of moving commodities down the supply chain, particularly to peripheral health facilities and CHWs, remain challenging and underfunded.

Introducing alternative ACTs to healthcare systems that are not sufficiently accessible and where the quality of care is inadequate could result in minimal impact on the spread and intensification of artemisinin and partner drug resistance. If newer ACTs are not consistently available, if these drugs are misused or over-prescribed, or if dosing is incorrect, it is likely that the development of resistance to the new partner drugs may be accelerated, and partial resistance to artemisinin may be further intensified.^12^

Much can be learned from the few countries that have successfully mitigated the effects of artemisinin and partner drug resistance. The best example comes from the most unlikely of countries. Partial resistance to artemisinin emerged as a public health concern in Cambodia in the early 2000s, particularly in the Thai–Cambodian border area.^13^ The first decade of response to this challenge focused on two areas: 1) monitoring resistance patterns through therapeutic efficacy studies and using these data to select the best ACT combinations and 2) implementing a containment strategy that targeted malaria control and elimination efforts in the border area and among mobile populations. By the early 2010s, partial artemisinin resistance had spread or arisen *de novo* throughout the Greater Mekong sub-region, and partner drug resistance also spread and intensified.

With support from the WHO, the PMI, the Global Fund, and other partners, Cambodia shifted its focus toward the goal of eliminating malaria from the entire country through an updated strategy.^14^ This strategy is based on expanding access to high-quality malaria diagnosis and treatment services in all endemic areas via a network of trained and supervised Village Malaria Workers; strengthening the malaria surveillance system, including the introduction of individual case reporting and investigation in low-prevalence areas; and expanding the use of surveillance data to identify and target high transmission areas, particularly forested and forest-fringe areas. Trained Mobile Malaria Workers equipped with rapid diagnostic tests and ACTs were also deployed to join groups entering high-risk forest areas for foraging, logging, or other purposes. Within a few years of implementing this approach, indigenous falciparum malaria transmission in the two main border provinces, Battambang and Pailin, was interrupted, with the rest of Cambodia quickly following suit. Cambodia appears to be close to interrupting the domestic transmission of falciparum malaria despite intense and widespread artemisinin and multiple partner drug resistance.

It would be naïve to believe that what was accomplished in Cambodia could be easily transferred to all countries in SSA, particularly given the differences in transmission intensity and dynamics, as well as the structures of the health delivery systems. However, no one could credibly dispute that prioritizing both the availability of alternative ACTs and expanded access to high-quality malaria diagnosis and treatment services was essential to Cambodia’s successful effort to mitigate drug resistance and eliminate malaria.

It should be acknowledged that nearly all malaria-affected countries, with support from the PMI, the Global Fund, and other funders and partners, have made efforts to expand access to and improve the quality of clinical care for malaria. However, with few exceptions, that support has not been sufficient to ensure that everyone at risk of malaria has access to high-quality and effective diagnosis and treatment.

Beyond the genuine need for available and affordable alternatives to current first-line ACTs, a renewed commitment to ensuring that these alternative ACTs are delivered through high-quality, accessible, and affordable health services must be prioritized. This will require the accelerated expansion of iCCM and QI schemes; the further strengthening of country supply chain systems with a focus on last-mile delivery strategies; and serious, sustained, and tailored efforts to develop and deploy QI strategies for private sector outlets, along with a strengthening of the country’s regulatory enforcement capacity.

There is no question that achieving these objectives will be a daunting task requiring new infusions of domestic and donor support, which is no small challenge given the stagnation of global malaria funding. However, there are opportunities to leverage support or collaborate with other global initiatives. For example, the objective of universal access to malaria case management services fully aligns with the goals and objectives of the UHC Movement, which was endorsed by a resolution of the 68th UN General Assembly in 2023.^15^ In addition, newer investments by countries and donors in pandemic preparedness in response to the coronavirus disease 2019 pandemic should focus on ensuring easy access to high-quality health services. Existing coordinated efforts with United States President’s Emergency Plan for AIDS Relief and the Global Fund to strengthen supply chains for HIV, tuberculosis, and malaria commodities could also be expanded, as could collaborations with programs and colleagues focused on enhancing reproductive, maternal, neonatal, and child health.

New, affordable, and accessible malaria treatments, including novel non-artemisinin-based options, are certainly needed. If the global malaria community does not want to issue another plea for newer affordable malaria treatments in the next 5–10 years because alternative ACTs are failing, a comprehensive approach to achieving universal access to high-quality malaria case management services must be implemented.

***Author’s note:*** *When this manuscript was submitted and accepted, the author could not have anticipated the recent freeze on US foreign assistance, including all support provided by PMI. It is hoped that this freeze will be quickly lifted so that support for life-saving drugs and malaria services can resume, and excess sickness and death from malaria can be averted.*
